# From Genes to Behavior: The Question of Evolutionary Conservation and the Role of Ethology in the Analysis of the Zebrafish

**DOI:** 10.3389/fnana.2021.809967

**Published:** 2021-12-02

**Authors:** Robert Gerlai

**Affiliations:** Department of Psychology, University of Toronto Mississauga, Mississauga, ON, Canada

**Keywords:** ethological relevance, evolutionary history, evolutionary homology, natural environment, translational relevance, zebrafish

## Introduction

Teleosts (ray-finned or bony fishes) represent the most species rich taxon among vertebrates. From the open pelagic regions of the Pacific Ocean to the underground caves of Mexico, these species have adapted to practically any habitat where liquid water exists on Earth (Nelson et al., 2016). The rich diversity of fish species represents a goldmine of information for scientists who seek to understand how biological characteristics evolve and how they relate to the features of the natural environment (Cossins and Crawford, 2005). Fish are a wonderful tool for comparative approaches. This opinion article, however, will focus on a single species, the zebrafish. The reason is simple. By now, the zebrafish has become the focus of numerous subfields of biology, and has become perhaps the most well-studied fish species especially in genetics and embryology (Grunwald and Eisen, 2002). In neurobiology, it is still a newcomer compared to traditional model organisms of biomedical research including rodents, but increasing amount of information is collected about its brain. Similarly, the number of studies investigating its behavioral characteristics is orders of magnitude less compared to those on rodents, but it is exponentially increasing (Kalueff et al., 2014). Thus, by now it is possible to employ multidisciplinary approaches combining a variety of genetics, neuroscience and behavioral methods to understand how its brain works.

## Translational Relevance

Perhaps the most important reason why this species has become preferred in biomedical research is that despite its relative simplicity it is argued to offer translationally relevant discoveries (Pickart and Klee, 2014). That is, discoveries made fast and cheaply with the zebrafish have been argued to help us understand the biological functioning and malfunctioning of other species, including our own. This is because the zebrafish is argued to possesses evolutionarily conserved features. It is a logical argument. We know the overwhelming majority, if not all, species on Earth are related to each other, i.e., at some point in the evolutionary past had a common ancestor. For fish and humans this was ~400 million years ago. In other words, the zebrafish and humans have been evolving separately for only 0.4 billion years whereas these two species share about 3.1 billion years of common biological evolution (assuming that life started about 3.5 billion years ago). As a result, there must be a large number of biological features that humans share with the zebrafish. But how do we tell what features are evolutionarily conserved? Is phenotypical similarity what we should be looking for?

## Evolutionary Homology vs. Analogy

The question of what is similar (and also what is different) between two species from an evolutionary standpoint may be best tackled within the framework of evolutionary homology vs. analogy. Biological structures and/or functions homologous across species are those that have a common evolutionary origin. These structures and functions may not necessarily appear similar at the phenotypical level. On the other hand, biological structures and/or functions may appear similar due to similar underlying natural selection forces, past environmental/ecological demands, yet they may have different evolutionary origins, a situation we call “analogy.” When we talk about translational relevance and we invoke the idea of evolutionarily conserved features, we are talking about evolutionary homology. This is because evolutionary homology means similarity at some level of underlying mechanisms, and to model and understand human conditions with laboratory animals we need this mechanistic similarity. Given that we do not have fossilized evidence on neurobiological structures and functions every step on the way in the lineages of species from the 0.4 billion-year-old common ancestor to modern day zebrafish and humans, ascertaining what represents evolutionary homology vs. analogy may not be a trivial task.

## The Level of Analysis

The complexity of how one can establish evolutionary homology depends upon the level of analysis, i.e., at what level of the biological organization one studies the organism. For scientists concerned with the question of how the brain works, I regard genes the bottom, most fundamental level, and behavior the highest level, with sub-disciplines of neuroscience studying everything in between. Establishing evolutionary conservation is relatively easy at the gene level. For example, a vast literature shows the nucleotide sequence of fish and human genes are similar enough to allow finding human orthologs with high confidence based on fish sequences and vice versa (Boffelli et al., 2004). Such nucleotide sequence similarities are unlikely to arise as a result of convergent (parallel) evolution, thus they represent evolutionary homology. One can also quantify the degree of similarity in synteny (chromosomal order of loci) between the zebrafish and human genomes (Barbazuk et al., 2000). Above the genetic level, however, measuring degree of similarity can become difficult. A small change in nucleotide sequence may induce dramatic structural/functional alteration of the translated protein that cannot be simply predicted from the nucleotide sequence alteration. Also, this alteration in turn can drastically modify the ontogenetic processes in which that protein is involved and also how the fully grown organism functions. A small nucleotide sequence difference may dramatically affect what phenotypical features and how the given protein variants will influence in one vs. another species.

## Behavioral Analysis and Its Role in Multidisciplinary Studies

The issues become even more complicated at the level of behavior, the ultimate output of the brain. Consider one example. The zebrafish is a social species. These fish form shoals, aggregates of individuals in which fish stay close to one another (Miller and Gerlai, 2011). Humans are highly social too. We organize ourselves into families, tribes, sports-clubs, nations. We stay physically and psychologically close to each other. Is high sociality of zebrafish an analogous or a homologous feature to how humans behave? Can we study the mechanisms of zebrafish social behavior and conclude anything about human social behavior and human diseases associated with abnormal social behavior? How do we know these behavioral phenomena are evolutionarily conserved and thus have common underlying mechanisms? Answering these questions cannot be obtained from behavioral analysis alone. It requires a multidisciplinary analysis of the biology of social behavior, from genes through biochemistry, synaptic and cellular functions, connectome and neuroanatomy, in addition to behavior. Admittedly, such multidisciplinary and systematic comparisons between fish and humans have not been performed. What we have got so far are small bits and pieces of sporadic info on certain apparent similarities between these two species. Whether these similarities represent evolutionary analogies or homologies is often unclear.

Neuroanatomy, although is above the level of genetics, does offer certain objective ways with which we can evaluate evolutionary homology vs. analogy, at least better than what we could accomplish with behavioral analysis alone. For example, although structurally the zebrafish and the human brain appears quite different, following the steps of brain development in these two species allows identification of homologous brain structures. Briefly, the fish brain develops via the process of eversion whereas the mammalian brain *via* evagination. These two developmental processes lead to distinct positioning of homologous structures, yet with preserved order and often similar cytoarchitecture and connectome as well as behavioral function. An interesting example of this is the lateral pallium of cyprinids (zebrafish belongs to the family of Cyprinidae), which is on the side of the fish forebrain, being homologous to the mammalian hippocampus, a deep subcortical structure part of the ancient limbic system (Broglio et al., 2010; Mueller, 2012). Both structures have been identified to be involved in complex forms of learning in these two sets of species: relational learning (Eichenbaum, 1992; Broglio et al., 2010; Karnik and Gerlai, 2012).

If evolutionary homologies are easier to identify at levels of analysis including genetics and neuroanatomy than at the level of behavior, should we study behavior at all? There are reasons why the answer to this question is yes. Behavior is the ultimate output of the brain, the endpoint of natural selection. It is also the endpoint of pre-clinical and clinical studies of human brain disorders. Furthermore, behavioral analysis helps us find functional alterations of the brain in an efficient manner (Gerlai, 2002). Thus, the question is not whether but how we should conduct behavioral analysis.

## Ethological Relevance

In the distant past, the debate centered around two distinct forms of analysis of behavior: the European school of ethology and North American animal psychology. The latter emphasized laboratory control and general aspects of behavior, the former taught that one must take species-specific aspects of behavior into account, i.e., one must not be nature blind. Nowadays, the amalgamation of the teachings of these two schools is what most accept as the best approach. Well-controlled laboratory experiments conducted in accordance with the evolutionary past and ecology of the studied species is the way to go (Gerlai and Clayton, 1999), i.e., laboratory behavioral analysis must be ethologically relevant. But what is ethological relevance and why is this important if we are to understand brain function?

It may appear paradoxical, but if we want to be able to properly translate findings obtained with zebrafish to human, we should know about the species-specific features of zebrafish, its ecology and natural behavior. This is because without such knowledge, the laboratory conditions we set up may be too artificial to yield meaningful results. Under artificial conditions, the zebrafish may not perceive, process or respond to stimuli appropriately. Its brain would face a task to which it has not adapted. Under artificial conditions, one may expect elevated stress, increased experimental error variance, and thus reduced statistical power to find effects of experimental manipulations significant (Gerlai and Clayton, 1999). But how do we tell what represents artificial conditions and what may be natural for the zebrafish in the laboratory? Let us examine an example to illuminate the problem.

Learning has been thoroughly studied in multiple species. The zebrafish too has been analyzed in this context (Gerlai, 2016, 2020a). A number of surprises have surfaced though. Although not well-documented in the literature, zebrafish learning studies struggled with a trivial problem. Food reward, a classic appetitive reinforcer, often did not work. The reason is that the fish satiated fast, and thus stopped working in the learning task (Gerlai, 2020b). In nature, zebrafish likely eat tiny amounts of insects sporadically throughout the day, unlike in the laboratory with bulky protein-rich fish-food offered as reinforcement. Knowing the natural eating habits of zebrafish and the food types they consume at large would help us develop proper reinforcement. Furthermore, most learning studies are conducted in novel experimental test apparati into which the test subject is placed by the experimenter. This has worked well for laboratory rodents, which have been domesticated, i.e., due to unintended artificial selection got adapted to human handling. The zebrafish is not such a species. Human handling induces robust antipredator responses in zebrafish (Tran and Gerlai, 2016), which makes it difficult to use the fish in learning tasks. Instead of trying to find the food reward, the fish freeze. Knowing the natural predators, the stimuli and contexts that induce fear responses in zebrafish, one should be able to ameliorate this problem. Last, zebrafish, just like rodents, are often tested in mazes or small tanks. Even their holding tanks are tiny, ranging between 1 and 3 liters. Rodents burrow and tunnel underground. They are used to small restricted spaces. In nature, zebrafish do not live in such restricted environments. They occupy the middle to the upper layer of water and are found in open areas near vegetation. Understanding their microhabitat preference would allow us to design proper holding and behavioral test tanks.

Designing naturalistic experimental test apparati and procedures will not be an easy task. It will require systematic and parametric analysis and characterization of the stimuli to which zebrafish respond, the motor reactions with which they respond and the motivational aspects of these responses. The analysis will also have to include both the physical aspects of the test environment and the procedural aspects of human-zebrafish interaction. Furthermore, I believe the answers to the above questions will be context (test paradigm) dependent. Although a simple recipe as to how to conduct behavioral studies with zebrafish may not be available, I believe the guiding principle of ethological relevance will bring fruitful answers to the above questions.

## Concluding Remarks

Comparing multiple fish species with different degree of relatedness living in a variety of habitats allows us to illuminate answers to both mechanistic and evolution related questions. Focusing on a single species like zebrafish has the advantage of being able to use powerful multidisciplinary studies. Comparing multidisciplinary results obtained for zebrafish and mammals, including our own species, is a way to establish translational relevance. Inclusion of behavioral analysis in such multidisciplinary work is important. Increasing our knowledge on the ecology and ethology of zebrafish will help us conduct behavioral studies and integrate their results into our research on mechanistic and evolutionary aspects of brain structure and function.

## Author Contributions

The author confirms being the sole contributor of this work and has approved it for publication.

## Funding

RG has been funded by NSERC Canada Discovery Grant (311637), the University of Toronto Mississauga Distinguished Professor Award and the University of Toronto Mississauga Provostial Research Grant.

## Conflict of Interest

The author declares that the research was conducted in the absence of any commercial or financial relationships that could be construed as a potential conflict of interest.

## Publisher's Note

All claims expressed in this article are solely those of the authors and do not necessarily represent those of their affiliated organizations, or those of the publisher, the editors and the reviewers. Any product that may be evaluated in this article, or claim that may be made by its manufacturer, is not guaranteed or endorsed by the publisher.
